# Effects of high-intensity interval training on aerobic and anaerobic capacity in olympic combat sports: a systematic review and meta-analysis

**DOI:** 10.3389/fphys.2025.1576676

**Published:** 2025-05-09

**Authors:** Fengshan Yue, Yuyan Wang, He Yang, Xiaolei Zhang

**Affiliations:** ^1^ Department of Physical Education, Northwestern Polytechnical University, Xi’an, China; ^2^ Public Teaching Department, Xinyang Aviation Vocational College, Xinyang, China; ^3^ School of Physical Education, University of Sanya, Sanya, China

**Keywords:** judo, boxing, wrestling, taekwondo, fencing, karate, intermittent exercise

## Abstract

This study aimed to summarize the findings of research comparing the effects of high-intensity interval training (HIIT) with active controls (alternative training methods) and passive controls (no exercise intervention) on aerobic and anaerobic performance in male and female athletes engaged in Olympic combat sports. Using the PICOS framework, the study included original research on healthy, trained male and female athletes in Olympic combat sports. These studies compared HIIT interventions (lasting at least 4 weeks) with control groups, focusing on aerobic and anaerobic performance outcomes. Studies that measured other fitness parameters, had non-randomized designs, or involved mixed interventions were excluded. A database search was conducted on February 1, 2025, through PubMed, Scopus, and Web of Science. Study quality and risk of bias were assessed using the Physiotherapy Evidence Database (PEDro) scale, while the GRADE scale was used to assess the certainty of evidence. After screening, 20 studies were deemed eligible for inclusion in this review. The results showed a significant effect of HIIT over control groups for maximal oxygen uptake, with a moderate effect size (ES = 1.007, 95% CI 0.701 to 1.312, p < 0.001). A significant, but small, effect favoring HIIT was also found for peak power output (ES = 0.528, 95% CI 0.102 to 0.954, p = 0.015). Furthermore, the analysis of mean power output showed a moderate significant effect of HIIT over controls (ES = 0.871, 95% CI 0.392 to 1.350, p < 0.001). In conclusion, HIIT, whether performed through running or sport-specific techniques, appears to effectively enhance both aerobic and anaerobic performance in athletes participating in Olympic combat sports. These improvements could contribute to better overall performance, supporting the physical and physiological demands of these sports.

## 1 Introduction

Both aerobic and anaerobic capacities are crucial for success in Olympic combat sports (i.e., boxing, wrestling, judo, taekwondo, karate, fencing), albeit to varying degrees depending on the specific discipline (Bridge et al., 2014; Franchini et al., 2014; Chaabène et al., 2015; Chaabene et al., 2017). Aerobic capacity can be essential for recovery between high-intensity bursts, maintaining technical proficiency throughout a bout, and mitigating fatigue which can lead to errors and decreased performance (Kamandulis et al., 2018). While not the primary energy source during explosive actions, a well-developed aerobic system allows athletes to recover faster between rounds or exchanges, enabling them to perform repeated high-power outputs (Kirk et al., 2024).

Additionally, anaerobic capacity can be paramount for delivering the explosive movements characteristic of combat sports, such as throws, strikes, and takedowns (Slimani et al., 2017). These high-intensity actions rely on the immediate energy provided by these anaerobic pathways (Franchini, 2020). Furthermore, the ability to repeatedly perform such high-power movements without significant decrement is vital, highlighting the importance of both alactic and lactic anaerobic capacity for maintaining performance consistency throughout a competition (Franchini, 2023). Therefore, optimizing both aerobic and anaerobic systems is crucial for combat sport athletes to maximize their performance potential (Franchini et al., 2019).

Aerobic conditioning traditionally involves moderate-intensity, prolonged activities that improve cardiovascular efficiency and the ability to sustain activity over time (Bridge et al., 2014; Franchini et al., 2014; Chaabène et al., 2015; Chaabene et al., 2017). However, aerobic training can also involve high-intensity, short-duration efforts, as seen in activities like high-intensity interval training (HIIT), which improves both cardiovascular endurance and the ability to clear metabolic byproducts during brief periods of exertion (Ruddock et al., 2021). In contrast, anaerobic conditioning targets high-intensity, short-duration efforts, enhancing the ability to produce energy through anaerobic pathways, thereby supporting explosive movements such as strikes, takedowns, or defensive actions (Franchini, 2023). These two forms of conditioning are complementary in combat sports, where athletes experience rapid shifts between sustained exertion and intense bursts of effort (Franchini, 2020). Interval training, which alternates between high-intensity bouts and recovery periods, can be particularly effective for developing both aerobic and anaerobic capacities.

HIIT may have critical importance for combat sports due to its ability to simultaneously enhance both aerobic and anaerobic performance (Ruddock et al., 2021). HIIT replicates the intermittent, high-intensity nature of combat sports, enabling athletes to train the precise energy systems demanded by their sport (Vasconcelos et al., 2020). From an aerobic perspective, HIIT improves cardiovascular fitness by increasing stroke volume and mitochondrial density (Rezaei et al., 2024). This allows athletes to sustain high levels of activity for longer periods of time (James et al., 2016). From an anaerobic perspective, HIIT may improve the ability to generate energy without oxygen (Herrera-Valenzuela et al., 2021). This is crucial for combat sports, as many of the explosive movements involved rely on anaerobic metabolism (Herrera-Valenzuela et al., 2021).

While HIIT is widely used in Olympic combat sports, a systematic review with meta-analysis is essential to definitively establish its efficacy and optimize its application. Prior reviews have been limited to summarizing the evidence in systematic reviews (Franchini et al., 2019; Vasconcelos et al., 2020), the last of which was completed in 2020. This necessitates an update to incorporate newer research. Furthermore, a meta-analysis, which quantitatively synthesizes data across studies, is critical to precisely determine the magnitude of HIIT’s impact on key aerobic and anaerobic performance measures. Such an analysis will enhance statistical power, providing more reliable estimates of HIIT’s effects than individual studies can offer. By pooling data, a meta-analysis can also resolve inconsistencies across studies and identify potential sources of heterogeneity. This comprehensive, evidence-based synthesis will empower coaches and athletes to design and implement more effective, sport-specific HIIT programs, ultimately maximizing athletic performance. Therefore, this study aims to summarize findings from studies comparing the effects of HIIT versus active controls (alternative training methods) or passive controls (no exercise intervention) on aerobic and anaerobic performance in male and female athletes participating in Olympic combat sports.

## 2 Methods

The guidelines outlined in the 2020 PRISMA (Preferred Reporting Items for Systematic Reviews and Meta-Analyses) Statement were followed in this systematic review (Page et al., 2021). Registered on February 03, 2025, the protocol for this systematic review is accessible on the Open Science Framework (registration number: osf.io/3njzg).

### 2.1 Eligibility criteria

The PICOS (Participants, Intervention, Comparator, Outcomes, Study Design) framework was used to determine eligibility. Original research articles published in peer-reviewed journals were included, with no restrictions placed on publication year (Rechenchosky et al., 2021) or language. The population consisted of healthy, trained/developmental male and female athletes of all ages, participating in any sport that has been part of the Olympic combat sports program (boxing, karate, wrestling, fencing, judo, or taekwondo). Excluded were injured athletes, para-athletes, and those in other sports. The intervention consisted of HIIT for at least 4 weeks, in various modalities (e.g., running, cycling, sport-specific) and regimens (short/long intervals, sub-maximal/maximal). Studies combining HIIT with other training (except regular combat sport training) were excluded. Comparators were control groups using parallel approaches (e.g., continuous training) or regular combat sport training. Studies using HIIT as part of the control intervention were excluded. Outcomes included aerobic (e.g., maximal oxygen uptake, capacity in progressive test) and anaerobic (e.g., maximal output, fatigue index) performance data measured at baseline and post-intervention. Studies with other physical fitness measures or acute responses were excluded. Multi-arm randomized controlled trials were included; quasi-experimental, descriptive, non-randomized studies, and reviews were excluded.

### 2.2 Information sources

We conducted a database search on February 01, 2025, using PubMed, Scopus, and Web of Science (Core Collection), in line with the registered protocol. Second, we manually examined the reference lists of all included studies to identify additional relevant publications. Third, we employed snowball citation tracking through the Web of Science database. Finally, we checked all selected studies for any associated errata or retractions.

### 2.3 Search strategy

To maximize the retrieval of relevant studies, Boolean operators “AND” and “OR” were strategically employed in the search strategy. No limitations were imposed regarding publication date, language, or study design, ensuring a comprehensive search. This approach aimed to capture all potentially relevant studies without narrowing the scope. The specific search methodology (Supplementary Material 1) is detailed below:

[Title/Abstract] box* OR wrestl* OR judo OR taekwondo OR karate OR fencing OR fencer*

AND

[Title/Abstract] “high-intensity interval training” OR “HIIT” OR “intermittent exercise” OR “sprint interval training” OR “repeated sprint training”

The initial search strategy did not incorporate a specific filter dedicated to outcomes, as the primary objective was to capture a broad spectrum of studies and maximize the number of potentially relevant articles for the first round of screening. By avoiding an overly restrictive search, we aimed to minimize the risk of prematurely excluding studies that might contain valuable information. A more refined or filtered search could have unintentionally led to the exclusion of relevant articles, potentially limiting the comprehensiveness of the review.

### 2.4 Selection process

Two authors (FY and YW) screened studies in the initial research phase, reviewing titles and abstracts. Abstracts of selected studies were then assessed against pre-defined inclusion criteria, and full-text articles were retrieved as necessary. Subsequently, the same two authors independently evaluated the full texts of studies that met the initial screening criteria. Disagreements at either stage were resolved through discussion. A third reviewer (XZ) was consulted if consensus could not be reached. Mendeley software, along with manual methods, facilitated record management and deduplication.

### 2.5 Data collection

FY initiated data extraction, which was then reviewed by YW and HY to ensure accuracy and completeness. A custom Microsoft Excel spreadsheet (Microsoft®, United States) was used for data capture. If full-text articles lacked data, FY contacted corresponding authors via email. Data from studies with no response after 1 week were excluded from the review and meta-analysis. Extracted data included: 1) sample size; 2) combat sport, age, and sex; and 3) study design details, including randomization. Training intervention data included: 1) duration and frequency; 2) total training sessions; and 3) the specific regimen (e.g., sets, repetitions, exercises, equipment). Moreover, HIIT training was classified based on the information from the studies as follows (Buchheit and Laursen, 2013): short HIIT (efforts >15 s per repetition; sub-maximal; rest duration <15 s), long HIIT (efforts > 2–3 min per set; sub-maximal; rest duration <2 min), repeated sprint training (RST; all-out efforts, >4 s per effort; rest duration <20 s), and sprint interval training (SIT; all-out efforts, >20 s per effort; rest duration >2 min).

### 2.6 Data items

Data were categorized as either aerobic or anaerobic measurements. Aerobic measurements encompassed, but were not limited to, direct or indirect assessments of maximal oxygen uptake, anaerobic threshold, maximal aerobic speed, or maximal aerobic capacity derived from progressive or specific-combat sport exercise tests to exhaustion (continuous or intermittent). Anaerobic measurements included, though were not limited to, assessments of maximal or mean power, or fatigue index.

### 2.7 Risk of bias assessment

The Physiotherapy Evidence Database (PEDro) scale, a reliable and effective tool validated for physiotherapy-based randomized clinical trials, was used to assess study quality and risk of bias assessment. The 11-item scale assesses key aspects of study design and reporting, such as random and concealed allocation, baseline comparability, blinding of participants and assessors, retention rates, intention-to-treat analysis, and outcome reporting. Each item is scored as “yes” (1) or “no” (0), with items 1 and 2 excluded from the total score (maximum 10). Quality is categorized as poor (≤3), fair (4–5), good (6–8), or excellent (≥9). Two authors (YW and HY) independently evaluated each study using the PEDro scale, resolving disagreements through discussion or, if needed, consultation with a third author (XZ).

### 2.8 Synthesis of results

For outcomes with data from at least three studies (i.e., aerobic and anaerobic), meta-analyses were conducted, regardless of the specific measurement. Primary outcomes’ Hedges’ g effect sizes (ES), 95% confidence intervals (CI), and 95% prediction intervals (PI) were calculated for both groups. Effect sizes were calculated from pre- and post-intervention means and standard deviations, standardized by post-intervention standard deviations. The DerSimonian and Laird random-effects model was used to account for inter-study variability and improve the reliability of overall findings, especially regarding small-study effects (SSE) (Deeks et al., 2008; Kontopantelis et al., 2013).

Effect sizes (ES) with 95% CIs were interpreted as: 0.0–0.2 (trivial), 0.2–0.6 (small), >0.6–1.2 (moderate), 1.2–2.0 (large), 2.0–4.0 (very large), and >4.0 (extremely large) (Hopkins et al., 2009). Control group sample sizes were proportionally adjusted in studies with multiple intervention groups. Heterogeneity was assessed with the I^2^ statistic (<25%: low, 25%–75%: moderate, >75%: high) (Higgins and Thompson, 2002). The extended Egger’s test (≥10 studies per outcome) assessed publication bias for continuous variables. If bias was found, a sensitivity analysis using the trim and fill method (L0 estimator) was performed. SPSS (version 29.0, IBM Corp., United States) was used for all analyses (p ≤ 0.05).

### 2.9 Certainty of evidence

FY and YW independently assessed the quality of evidence using the GRADE (Grading of Recommendations Assessment, Development, and Evaluation) approach. Their evaluation considered four of the five core GRADE criteria: risk of bias, inconsistency, imprecision, and publication bias. Randomized controlled trial evidence was initially considered high-certainty. This level could be adjusted based on the assessment criteria. For example, high risk of bias, inconsistency of results, or indirect evidence would lead to downgrading, while a large effect size or a dose-response gradient would result in upgrading. The final certainty of evidence was categorized into four levels (high, moderate, low, or very low), representing the confidence in the estimated effect.

## 3 Results

### 3.1 Study selection

**FIGURE 1:**
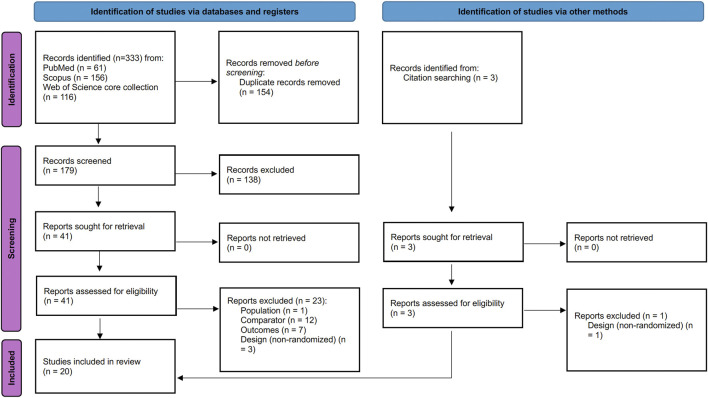
PRISMA flowchart (Page et al., 2021).

A total of 333 studies were initially identified through a search across the databases. After duplicates were removed (n = 154), 179 records remained and were screened based on their titles and abstracts. Then, 138 studies were excluded, leaving 41 studies for full-text analysis. After this review, 19 studies were excluded for not meeting eligibility criteria: 1 was excluded for not meeting the population criteria, 12 for not meeting the control group, 6 for not meeting the required outcomes, and 3 for not meeting randomization. The full list of included and excluded studies, along with the reasons for exclusion, can be found in Supplementary Material 2. Therefore, 18 studies were deemed eligible, and following manual citation searches, 2 additional eligible articles were identified, bringing the total number of studies included in the systematic review to 20, as shown in Figure 1.

### 3.2 Study characteristics

Table 1 summarizes 20 studies examining the effects of HIIT across Olympic combat sports. Judo was the most frequently studied discipline, represented in 7 studies, closely followed by Taekwondo with 6. Wrestling appeared in 2 studies, as well as Boxing and Karate with 2 studies each. Regarding participant sex, 12 studies included only male athletes, while 7 studies incorporated both male and female participants. The standardized mean age of participants across all studies, weighted by the number of participants, is approximately 20.3 years. The ages of the participants in these studies ranged from a minimum of 15.0 years to a maximum of 27.9 years. Across the studies included, a total of 399 participants were examined. Of these, 199 athletes participated in HIIT interventions, while the remaining 200 served as controls.

**TABLE 1:** Summary of study characteristics: design, population, intervention, and outcomes.

Study	Combat sport	Age (y)	Sex	HIIT (n)	Control (n)	Duration (w)	Sessions (n)	HIIT type	HIIT protocol	Control	Outcomes
Batra and Zatoń (2016)	Taekwondo	24.5 ± 4.1	Men	10	10	8	16	SIT	2 sets of 4–6 reps, each rep consisting of 30 s of maximal kicking drills and 90 s of rest, for a total session time of 45 min	Continued their regular training routnes	Aerobic: VO2max (ml/kg/min) Anaerobic: VE/VO2 (l/min) and VE/VCO2 (l/min)
Farzad et al. (2011)	Wrestling	19.8 ± 2.2	Men	8	7	4	8	RST	3–6 sets of 6 × 35-m sprints, with each sprint followed by a 10-s rest and each set followed by a 3-min rest	Continued their regular training routnes	Aerobic: VO2max (ml/kg/min) Anaerobic: PPO (W); MPO (W)
Franchini et al. (2016b)	Judo	23.9 ± 5.9	Men	9^a^ 9^b^ 9^c^	8	4	8	RST	^a^: 2 sets of 10 sets of 20 s of all-out cycling at 4.5% of body mass resistance, with 10 s rest between sets and 5 min rest between blocks ^b^: 2 sets of 10 sets of 20 s of all-out upper-body cycling at 3% of body mass resistance, with 10 s rest between sets and 5 min rest between blocks ^c^: 2 sets of 10 sets of 20 s of all-out uchi-komi (technique entrance and throw), with 10 s rest between sets and 5 min rest between blocks	Continued their regular training routnes	Aerobic: VO2peak (L/min) Anaerobic: total work (kj)
Franchini et al. (2016a)	Judo	23.9 ± 5.9	Men	9^a^ 9^b^ 9^c^	8	4	8	RST	^a^: 2 blocks of 10 sets of 20 s all-out cycling at 4.5% body mass resistance, with 10 s rest between sets and 5 min rest between blocks ^b^: 2 blocks of 10 sets of 20 s all-out upper-body cycling at 3% body mass resistance, with 10 s rest between sets and 5 min rest between blocks ^c^: 2 blocks of 10 sets of 20 s all-out uchi-komi (technique entrance and throw), with 10 s rest between sets and 5 min rest between blocks	Continued their regular training routnes	Anaerobic: SJFT index (bpm/throws)
Guo and Mu (2024)	Wrestling	19.7 ± 0.8	Men	10^d^ 10^e^	10	7	21	SIT	Both the SIT^d^ and SIT^e^ groups performed 4 sets of 5-s all-out runs with a 1:3 work-to-rest ratio (5 s all-out, 15 s self-paced recovery), and a 3-min active rest between sets. SIT^d^ group progressed from 6 repetitions per set in week 1–12 repetitions per set in week 7, while the SIT^e^ group maintained 9 repetitions per set throughout the 7-week program	Continued their regular training routnes	Aerobic: VO2max (ml/kg/min) Anaerobic: PPO (W); MPO (W)
Herrera-Valenzuela et al. (2021)	Boxing	27.9 ± 6.6	Men	8	8	4	12	RST	3 blocks of 5 repetitions of 30-s punching bag work with 6 s rest between repetitions and 1 min rest between blocks	Continued their regular training routnes	Aerobic: VO2max (ml/kg/min)
Kamandulis et al. (2018)	Boxing	21.9 ± 1.3	Men	9	9	4	12	SIT	3 rounds of 14 sets of 3-s all-out punching with 10 s rest between punches and 1 min rest between rounds	Hit the punching bag for 3 rounds of 3 min at low intensity, with 1-min rest	Aerobic: VO2max (ml/kg/min) Anaerobic: PPO (W)
Kim et al. (2011)	Judo	20.0 ± 1.1	Men	11	18	8	32	SIT	6 sprints in weeks 1–2, 8 sprints in weeks 3–4, and 10 sprints in weeks 5–8. Each sprint was 30 s at 80% MAV (weeks 1–2) and 90% MAV (weeks 3–8), with a 4-min warm-up and 4 min of recovery between sprints	Continued their regular training routnes	Aerobic: VO2max (ml/kg/min) Anaerobic: PPO (W); MPO (W)
Lee et al. (2015)	Judo	20.0 ± 1.0	Men	10	9 10^f^	12	48	short HIIT	6 sprints (weeks 1–2), 8 sprints (weeks 3–8), and 10 sprints (weeks 9–12). Each sprint was 30 s at 80% MAV (weeks 1–2) and 90% MAV (weeks 3–12), with a 4-min warm-up and 4 min of recovery between sprints	One group: continued their regular training routnes Second group: performance resistance training	Aerobic: VO2max (ml/kg/min) Anaerobic: PPO (W); MPO (W)
Monks et al. (2017)	Taekwondo	18 to 22	Both	16	17^g^	4	11	short HIIT	Weeks 1 and 2 involve 3 sessions per week, each with 3 sets of intervals. Week 3 reduces to one session with the same 3-set structure, and two sessions with a single set of more varied intervals. Week 4 consists of two sessions, each again with 3 sets of varied intervals. Work intervals range from 5 s to 60 s, 85%–100% HRmax with corresponding rest periods	Performed continuous running at 85%HRmax	Aerobic: VO2max (ml/kg/min) Anaerobic: PPO (W); MPO (W)
Ojeda-Aravena et al. (2019)	Karate	15.6 ± 2.4	Men	3^h^ 3^i^	3	6	18	SIT	6–8 sets of 30-s all-out efforts (countermovement jumps for HIIT-JUMP^h^, 5 m roundtrip sprints for HIIT-SPRINT^i^) followed by 2.5 min of rest, with the set volume decreasing to 6 in the final 2 weeks	Continued their regular training routnes	Aerobic: MSSR (min)
Ojeda-Aravena et al. (2021)	Taekwondo	19.5 ± 4.5	Both	8	8	4	12	SIT	3 rounds of high-intensity (RPE 10) alternating roundhouse kicks with varying repetitions and rest periods: Week 1–4 reps with 28s rest; Week 2–5 reps with 24s rest; Week 3–5 reps with 20s rest; Week 4–6 reps with 16s rest. All rounds had 1 min of active recovery between them	10 min of technical kicking with speed paddles and simulated sparring at moderate intensity	Aerobic: MSSR (m) Anaerobic: KDI (%)
Ouergui et al. (2020)	Judo	16.0 ± 1.0	Both	12^j^ 12^k^	12	4	8	RST	3 sets of 10 repetitions in week 1, increasing by one set per week to reach 6 sets by week 4. RST^j^ consisted of 35 m sprints with 10s passive rest, while RTT^k^ involved maximal repetitions of Bandal-tchagui kicks with 10s passive rest. There was a 3-min rest between sets	Continued their regular training routnes	Aerobic: VO2max (ml/kg/min) Anaerobic: fatigue index in repeated 5-m shuttle run (%)
Ouergui et al. (2022)	Taekwondo	15.0 ± 1.0	Both	14^l^ 16^m^ 19^n^	12	4	16	RST	The intensified training consisted of two blocks of 10 sets of 20-s all-out efforts with 10-s rests between sets and 5 min between blocks. During tapering, the HIIT sessions were reduced to one block	Continued their regular training routnes	Anaerobic: SJFT index (bpm/throws)
Rezaei et al. (2024)	Karate	20.0 ± 1.0	Both	8	8	6	18	Short HIIT	6–8 sets of 30-s high-intensity intervals (80%–100% HRmax) with 2.5 min of rest between sets. The set volume progressed from 6 to 8 sets and then back to 6 sets in the final 2 weeks	Continued their regular training routnes	Aerobic: VO2max (ml/kg/min) Anaerobic: PPO (W); MPO (W)
Seo et al. (2019)	Taekwondo	16.7 ± 0.8	Men	12° 12^p^ 12^q^	11	4	10	SIT	6–8 bouts of 30-s sprints at 90%–100% HRmax, with active recovery periods of 60s (1:2 ratio), 120s (1:4 ratio), or 240s (1:8 ratio) between bouts	Continued their regular training routnes	Aerobic: VO2max (ml/kg/min) Anaerobic: PPO (W); MPO (W)
Song and Sheykhlouvand (2024)	Taekwondo	19.8 ± 1.3	Men	10^j^ 10^r^	10	6	18	SIT	3 sets of 10 x 4-s all-out efforts (kicks for HIITTS^r^, running for HIITRS^j^) with 15 s of passive recovery between efforts and 1 min rest between sets	Continued their regular training routnes	Aerobic: VO2max (ml/kg/min) Anaerobic: PPO (W); MPO (W)
Tapia et al. (2020)	Taekwondo	20.8 ± 5.5	Both	6	6	4	12	RST	3 blocks of 6 repetitions of 10-s all-out bandal tchagui kicks, with 10 s of passive rest between repetitions and 1 min of rest between blocks	Continued their regular training routnes	Anaerobic: KDI (%)
Uchoa et al. (2020)	Judo	27.1 ± 8.2	Both	10	10	6	18	RST	Week 1–8 repetitions of Osoto-gari; Weeks 2–3–10 repetitions (5 Osoto-gari, 5 Ippon-seoi-nage); Weeks 4–6–12 repetitions (6 Osoto-gari, 6 Ippon-seoi-nage). Each repetition consisted of 30 s of effort and 15 s of rest	Continued their regular training routnes	Anaerobic: SJFT index (bpm/throws)
Zhang et al. (2024)	Judo	19.9 ± 0.7	Men	16^s^ 16^t^	16	6	12	RST	Two sets of high-intensity efforts. The 2:1 group performed 9 repetitions of 20-s efforts with 10-s rests, while the 3:1 group performed 6 repetitions of 30-s efforts with 10-s rests	Continued their regular training routnes	Aerobic: VO2max (ml/kg/min) Anaerobic: PPO (W); MPO (W)

^a^ : lower body training.

^b^ : upper-body training.

^c^ : Uchi-komi training.

^d^ : progressed HIIT.

^e^ : non-progressed HIIT.

^f^ : judo training + resistance training.

^g^ : High-intensity continuous exercise.

^h^ : HIIT, jump.

i : sprint interval training.

j : repeated sprint training.

^k^ : repeated technique training.

^l^ : kumi-kata.

^m^ : uchi-komi.

^n^ : running.

^o^ ; 1:2 ratio.

^p^ ; 1:4 ratio.

^q^ : 1:8 ratio.

^r^ : technique specific.

^s^ ; 3:1 ratio.

^t^ : 2:1 ratio.

VO2max, maximal oxygen uptake; VO2peak, peak oxygen consumption; VE/VO2, ventilatory equivalent for oxygen; VE/VCO2, ventilatory equivalent for carbon dioxide; PPO, peak power output; MPO, mean power output; SJFT, special judo fitness test; MSSR, multistage shuttle run; KDI, kick decreased index; RPE, rating of perceived exertion; SIT, sprint interval training; RST, repeated sprint training.

The most common study duration was 4 weeks, although study lengths ranged from 4 to a maximum of 12 weeks. The total number of training sessions ranged from a minimum of 8 to a maximum of 48, with 8 sessions appearing most frequently. Weekly training frequency averaged 2.68 sessions per week, with individual studies implementing between 2 and 4 sessions per week.

The VO2max, i.e., highest amount of oxygen the body can utilize during intense, sustained exercise (or a similar measure like VO2peak, i.e., highest oxygen consumption achieved during a specific exercise test, which may not necessarily be the maximal level the individual could reach), peak power output (PPO), and mean power output (MPO) were the most frequently assessed outcomes. Of the 20 studies, 14 reported VO2max (or a similar measure like VO2peak), 10 reported PPO, and 10 reported MPO.

### 3.3 Methodological quality of the studies

A total of 20 studies were assessed using the PEDro scale (Table 2). Of those, 19 studies can be considered to have “good” methodological quality, scoring 6 to 8, while only 1 study achieved a score of 9, representing “excellent” quality. The primary sources of bias across the included studies are related to blinding. Specifically, criteria C5 (blinding of all subjects) and C6 (blinding of therapists) were frequently unmet (n = 19 not reported), indicating a lack of blinding in most of studies. Additionally, C7, which assesses blinding of all assessors, was also commonly scored as zero (n = 17).

**TABLE 2:** Methodological quality assessment using the Physiotherapy Evidence Database scale (PEDro).

Study	C1	C2	C3	C4	C5	C6	C7	C8	C9	C10	C11	Score	Quality
Batra and Zatoń (2016)	0	1	0	1	0	0	0	1	1	1	1	6	Good
Farzad et al. (2011)	1	1	0	1	0	0	1	1	1	1	1	7	Good
Franchini et al. (2016b)	1	1	0	1	0	0	0	1	1	1	1	6	Good
Franchini et al. (2016a)	1	1	0	1	0	0	0	1	1	1	1	6	Good
Guo and Mu (2024)	1	1	0	1	0	0	0	1	1	1	1	6	Good
Herrera-Valenzuela et al. (2021)	1	1	0	1	0	0	0	1	1	1	1	6	Good
Kamandulis et al. (2018)	0	1	0	1	0	0	0	1	1	1	1	6	Good
Kim et al. (2011)	0	1	0	1	0	0	0	1	1	1	1	6	Good
Lee et al. (2015)	1	1	0	1	0	0	0	1	1	1	1	6	Good
Monks et al. (2017)	0	1	0	1	0	0	0	1	1	1	1	6	Good
Ojeda-Aravena et al. (2019)	0	1	0	1	0	0	1	1	1	1	1	7	Good
Ojeda-Aravena et al. (2021)	1	1	0	1	1	1	1	1	1	1	1	9	Excellent
Ouergui et al. (2020)	1	1	0	1	0	0	0	1	1	1	1	6	Good
Ouergui et al. (2022)	1	1	0	1	0	0	0	1	1	1	1	6	Good
Rezaei et al. (2024)	1	1	0	1	0	0	0	1	1	1	1	6	Good
Seo et al. (2019)	1	1	0	1	0	0	0	1	1	1	1	6	Good
Song and Sheykhlouvand (2024)	1	1	0	1	0	0	0	1	1	1	1	6	Good
Tapia et al. (2020)	0	1	0	1	0	0	0	1	1	1	1	6	Good
Uchoa et al. (2020)	1	1	0	1	0	0	0	1	1	1	1	6	Good
Zhang et al. (2024)	1	1	0	1	0	0	0	1	1	1	1	6	Good

C1: eligibility criteria were specified; C2: subjects were randomly allocated to groups; C3: allocation was concealed; C4: the groups were similar at baseline regarding the most important prognostic indicators; C5: there was blinding of all subjects; C6: there was blinding of all therapists who administered the therapy; C7: there was blinding of all assessors who measured at least one key outcome; C8: measures of at least one key outcome were obtained from more than 85% of the subjects initially allocated to groups; C9: all subjects for whom outcome measures were available received the treatment or control condition as allocated, or, where this was not the case, data for at least one key outcome were analyzed according to “intention to treat”; C10: the results of between-group statistical comparisons are reported for at least one key outcome; C11: the study provides both point measures and measures of variability for at least one key outcome.

### 3.4 Summary of the results

The studies summarized in Table 3 examined the HIIT on aerobic capacity in various groups. Some studies found significant improvements in VO2max post-HIIT compared to pre-HIIT values (Farzad et al., 2011; Batra and Zatoń, 2016; Ouergui et al., 2020, 2022; Herrera-Valenzuela et al., 2021; Guo and Mu, 2024), with few reporting HIIT being better than control groups (Kamandulis et al., 2018; Guo and Mu, 2024; Song and Sheykhlouvand, 2024; Zhang et al., 2024). Conversely, Franchini et al. (2016b) and Kim et al. (2011) found no significant differences within or between groups.

**TABLE 3:** Summary of the aerobic results.

Study	Outcome	HIIT (n)	HIIT mean (baseline)	HIIT SD (baseline)	HIIT mean (post)	HIIT SD (post)	Control (n)	Control mean (baseline)	Control SD (baseline)	Control mean (post)	Control SD (post)	Main findings
Batra and Zatoń (2016)	VO2max	10	50.1	3.8	53.2	2.5	10	53.5	2.1	53.7	2.9	post > pre HIIT (*p* = 0.002); no between-group difference
Farzad et al. (2011)	VO2max	8	49.3	4.4	52.0	3.4	7	51.2	6.1	50.1	4.7	post > pre HIIT (*p* = 0.002); no between-group difference
(Franchini et al., 2016b)^a^	VO2peak^f^	9	3.62	0.50	3.68	0.80	8	3.56	0.49	3.54	0.74	No significant differences within- and between-groups
(Franchini et al., 2016b)^b^	VO2peak^f^	9	3.82	0.59	3.86	0.44	8	3.56	0.49	3.54	0.74	No significant differences within- and between-groups
(Franchini et al., 2016b)^c^	VO2peak^f^	9	3.87	0.44	3.74	0.36	8	3.56	0.49	3.54	0.74	No significant differences within- and between-groups
(Guo and Mu, 2024)^d^	VO2max	10	48.9	1.0	51.3	1.1	10	48.6	1.4	48.5	1.2	post > pre HIIT (*p* = 0.001); HIIT > control (p = 0.048)
(Guo and Mu, 2024)^e^	VO2max	10	48.7	1.2	51.6	2.6	10	48.6	1.4	48.5	1.2	post > pre HIIT (*p* = 0.007); no between-group difference
Herrera-Valenzuela et al. (2021)	VO2max	8	46.5	5.1	48.6	5.6	8	47.6	3.8	48.0	2.6	Small magnitude of differences (ES = 0.42); no between-group difference
Kamandulis et al. (2018)	VO2max	9	33.1	1.2	40.7	2.3	9	35.0	1.2	35.2	1.5	post > pre HIIT (*p <* 0.005); HIIT > control (p < 0.005)
Kim et al. (2011)	VO2max	11	49.8	4.1	53.1	4.8	18	46.6	5.0	50.1	4.3	No significant differences within- and between-groups
Lee et al. (2015)	VO2max	10	49.9	4.3	54.4	4.3	9	46.6	2.6	48.7	2.6	HIIT > control (p = 0.03)
Monks et al. (2017)	VO2max	16	56.1	1.4	60.8	1.6	17	51.5	1.3	52.4	1.5	post > pre HIIT (*p <* 0.005); no between-group difference
(Ouergui et al., 2020)^g^	VO2max	12	39.6	3.6	44.2	3.9	12	37.2	4.9	36.8	5.4	post > pre HIIT (*p <* 0.005); HIIT > control (p < 0.005)
(Ouergui et al., 2020)^h^	VO2max	12	39.8	3.1	45.4	4.2	12	37.2	4.9	36.8	5.4	post > pre HIIT (*p <* 0.005); HIIT > control (p < 0.005)
Rezaei et al. (2024)	VO2max	8	50.6	6.9	57.2	3.9	8	46.0	5.1	46.1	3.0	post > pre HIIT (*p <* 0.005)
(Seo et al., 2019)^i^	VO2max	12	63.6	8.1	68.9	8.7	11	63.0	5.5	62.9	6.5	post > pre HIIT (*p <* 0.005)
(Seo et al., 2019)^j^	VO2max	12	66.5	5.5	70.9	7.5	11	63.0	5.5	62.9	6.5	post > pre HIIT (*p <* 0.005)
(Seo et al., 2019)^k^	VO2max	12	63.2	3.2	66.7	4.1	11	63.0	5.5	62.9	6.5	post > pre HIIT (*p <* 0.005)
(Song and Sheykhlouvand, 2024)^g^	VO2max	10	48.8	2.5	51.9	2.8	10	46.6	2.9	47.1	3.2	post > pre HIIT (*p <* 0.005); HIIT > control (p < 0.005)
(Song and Sheykhlouvand, 2024)^l^	VO2max	10	46.4	3.0	48.5	3.2	10	46.6	2.9	47.1	3.2	post > pre HIIT (*p <* 0.005)
(Zhang et al., 2024)^m^	VO2max	16	44.6	5.3	47.1	5.6	16	40.0	6.8	40.4	5.5	post > pre HIIT (*p <* 0.005); HIIT > control (p < 0.005)
(Zhang et al., 2024)^n^	VO2max	16	42.0	4.8	46.1	4.5	16	40.0	6.8	40.4	5.5	post > pre HIIT (*p <* 0.005); HIIT > control (p < 0.005)
(Ojeda-Aravena et al., 2019)^o^	MSSR	3	8#	6-10#	8#	6-9#	3	7#	5-7#	7#	4-9#	-
(Ojeda-Aravena et al., 2019)^p^	MSSR	3	8#	6-10#	8#	8-9#	3	7#	5-7#	7#	4-9#	-
Ojeda-Aravena et al. (2021)	MSSR	8	7.8	2.6	8.8	2.5	8	8.0	2.1	9.2	2.0	No significant differences within- and between-groups

VO2max: maximal oxygen uptake; MSSR: multistage shuttle run

^a^ : lower body training.

^b^ : upper-body training.

^c^ : Uchi-komi training.

^d^ : progressed HIIT.

^e^ : non-progressed HIIT.

^f^ : results from lower-body maximal graded test.

^g^ : repeated sprint training.

^h^ : repeated technique training.

^i^ ; 1:2 ratio.

^j^ ; 1:4 ratio.

^k^ : 1:8 ratio.

^l^ : technique specific.

^m^ ; 3:1 ratio.

^n^ : 2:1 ratio.

^o^ : HIIT jump.

^p^ : sprint interval training.

#:data represents median and 25 and 75 percentiles.

Table 4 summarizes the studies examining the effects of HIIT on Peak Power Output (PPO), mean power output (MPO), and other anaerobic measures. Most studies (Farzad et al., 2011; Kim et al., 2011; Monks et al., 2017; Kamandulis et al., 2018; Seo et al., 2019; Guo and Mu, 2024) report significant improvements in both PPO and MPO post-HIIT, with HIIT being better than control groups in also most cases (Farzad et al., 2011; Monks et al., 2017; Kamandulis et al., 2018; Seo et al., 2019; Guo and Mu, 2024). However, Lee et al. (2015), Seo et al. (2019), and Zhang et al. (2024) found no significant differences between the HIIT and control groups, nor any significant improvements within the HIIT groups.

**TABLE 4:** Summary of the anaerobic results.

Study	Outcome	HIIT (n)	HIIT mean (baseline)	HIIT SD (baseline)	HIIT mean (post)	HIIT SD (post)	Control (n)	Control mean (baseline)	Control SD (baseline)	Control mean (post)	Control SD (post)	Main findings
Farzad et al. (2011)	PPO#	8	972.4	252.4	1183.6	421.0	7	955.9	82.1	884.6	52.0	post > pre HIIT (p < 0.05); HIIT > control (p < 0.05)
(Guo and Mu, 2024)^d^	PPO	10	806.9	38.8	879.6	39.1	10	805.2	48.3	808.4	50.7	post > pre HIIT (*p =* 0.001); HIIT > control (p = 0.001)
(Guo and Mu, 2024)^e^	PPO	10	807.9	59.7	883.6	57.0	10	805.2	48.3	808.4	50.7	post > pre HIIT (*p =* 0.001); HIIT > control (p = 0.001)
Kamandulis et al. (2018)	PPO	9	176.3	11.1	187.4	5.9	9	196.4	12.8	193.0	7.3	post > pre HIIT (*p <* 0.005); HIIT > control (p < 0.005)
Kim et al. (2011)	PPO	11	12.8	1.3	15.5	1.7	18	12.9	1.9	13.9	2.3	post > pre HIIT (*p <* 0.01)
Lee et al. (2015)	PPO	10	12.9	1.4	14.4	1.2	9	12.4	2.1	13.2	2.1	No significant differences within- and between-groups
Monks et al. (2017)	PPO	16	710.6	29.8	762.2	26.9	17	695.7	28.8	693.7	25.8	HIIT > control (p < 0.05)
Rezaei et al. (2024)	PPO	8	10.5	3.5	11.8	3.1	8	9.2	1.9	9.7	2.3	post > pre HIIT (*p <* 0.005); no between-group difference
(Seo et al., 2019)^g^	PPO	12	689.6	123.9	692.1	101.2	11	709.8	98.6	712.4	83.4	No significant differences within-group
(Seo et al., 2019)^h^	PPO	12	702.2	144.1	775.6	121.3	11	709.8	98.6	712.4	83.4	HIIT > control (p < 0.05)
(Seo et al., 2019)^i^	PPO	12	699.7	60.7	780.6	80.9	11	709.8	98.6	712.4	83.4	HIIT > control (p < 0.05)
(Song and Sheykhlouvand, 2024)^p^	PPO	10	911.0	143.0	995.0	168.0	10	899.0	111.0	958.0	129.0	HIIT > control (p < 0.05)
(Song and Sheykhlouvand, 2024)^j^	PPO	10	861.0	119.0	919.0	122.0	10	899.0	111.0	958.0	129.0	HIIT > control (p < 0.05)
(Zhang et al., 2024)^k^	PPO	16	9.0	1.3	9.6	1.2	16	9.9	0.9	9.6	1.4	HIIT > control (p < 0.05)
(Zhang et al., 2024)^l^	PPO	16	9.0	0.8	9.1	0.9	16	9.9	0.9	9.6	1.4	No significant differences within- and between-groups
Farzad et al. (2011)	MPO	8	464.3	82.3	490	84.4	7	325	11.5	325.4	15	post > pre HIIT (p < 0.05); HIIT > control (p < 0.05)
(Guo and Mu, 2024)^d^	MPO	10	471.4	24.7	510.5	29.9	10	470.6	43.2	471.1	44.7	post > pre HIIT (*p =* 0.001); HIIT > control (p = 0.007)
(Guo and Mu, 2024)^e^	MPO	10	472.5	20.4	511.7	19.6	10	470.6	43.2	471.1	44.7	post > pre HIIT (*p =* 0.001); HIIT > control (p = 0.031)
Kim et al. (2011)	MPO	11	9.4	1.1	12.1	2.1	18	9.2	1.7	9.7	3.2	post > pre HIIT (*p <* 0.05)
Lee et al. (2015)	MPO	10	9.5	1.1	13.0	3.5	9	9.4	1.9	9.4	1.9	No significant differences within- and between-groups
Monks et al. (2017)	MPO	16	478.5	21.4	534.8	18.4	17	461.1	20.4	478.5	16.5	HIIT > control (p < 0.05)
Rezaei et al. (2024)	MPO	8	4.3	0.9	3.7	1.8	8	3.4	1.7	2.9	2.0	post > pre HIIT (*p <* 0.005); no between-group difference
(Seo et al., 2019)^g^	MPO	12	494.6	39.7	528.6	62.5	11	505.9	60.6	505.9	11.4	HIIT > control (p < 0.05)
(Seo et al., 2019)^h^	MPO	12	507.8	96.5	568.4	102.1	11	505.9	60.6	505.9	11.4	HIIT > control (p < 0.05)
(Seo et al., 2019)^i^	MPO	12	496.5	47.3	566.5	53.0	11	505.9	60.6	505.9	11.4	HIIT > control (p < 0.05)
(Song and Sheykhlouvand, 2024)^f^	MPO	10	590.0	65.0	680.0	82.0	10	611.0	59.0	643.0	51	HIIT > control (p < 0.05)
(Song and Sheykhlouvand, 2024)^j^	MPO	10	587.0	44.0	659.0	50.0	10	611.0	59.0	643.0	51	HIIT > control (p < 0.05)
(Zhang et al., 2024)^k^	MPO	16	6.6	1.0	7.1	0.9	16	7.0	0.8	7.1	0.7	HIIT > control (p < 0.05)
(Zhang et al., 2024)^l^	MPO	16	6.6	0.6	6.7	0.6	16	7.0	0.8	7.1	0.7	No significant differences within- and between-groups
(Franchini et al., 2016a)^a^	SJFT index	9	12.7	2.0	12.0	1.8	8	13.1	1.5	13.8	1.1	No significant differences within- and between-groups
(Franchini et al., 2016a)^b^	SJFT index	9	12.8	2.6	12.1	1.4	8	13.1	1.5	13.8	1.1	No significant differences within- and between-groups
(Franchini et al., 2016a)^c^	SJFT index	9	12.8	1.5	12.1	1.4	8	13.1	1.5	13.8	1.1	post > pre HIIT (*p <* 0.005)
Uchoa et al. (2020)	SJFT index	10	15.4	1.8	13.8	1.6	10	16.7	1.7	16.1	1.3	post > pre HIIT (*p <* 0.05); HIIT > control (p < 0.05)
(Ouergui et al., 2022)^m^	SJFT index	14	14.5	2.5	13.1	1.3	12	17.4	1.8	15.6	0.8	No significant differences within- and between-groups
(Ouergui et al., 2022)^n^	SJFT index	16	16.0	1.4	14.4	1.4	12	17.4	1.8	15.6	0.8	No significant differences within- and between-groups
(Ouergui et al., 2022)^o^	SJFT index	19	16.7	2.2	14.5	1.6	12	17.4	1.8	15.6	0.8	No significant differences within- and between-groups
Ojeda-Aravena et al. (2021)	KDI	8	7.9	3.9	5.2	3.2	8	11.1	4.3	7.1	2.4	No significant differences within- and between-groups
Tapia et al. (2020)	KDI	6	7.7	4.9	5.6	2.7	6	7.6	2.5	10.0	2.9	post > pre HIIT (*p <* 0.05)
Batra and Zatoń (2016)	VE/VO2	10	37.4	2.6	37.0	2.5	10	37.6	4.3	37.6	4.2	No significant differences within- and between-groups
Batra and Zatoń (2016)	VE/VCO2	10	33.0	2.8	33.5	2.6	10	34.5	4.0	34.4	33.0	No significant differences within- and between-groups
(Franchini et al., 2016b)^a^	total work	9	56.6	12.0	66.3	10.8	8	66.2	11.5	63.4	11.0	No significant differences within- and between-groups
(Franchini et al., 2016b)^b^	total work	9	64.6	6.8	61.0	10.2	8	66.2	11.5	63.4	11.0	No significant differences within- and between-groups
(Franchini et al., 2016b)^c^	total work	9	61.0	7.2	66.3	7.9	8	66.2	11.5	63.4	11.0	No significant differences within- and between-groups
(Ouergui et al., 2020)^f^	fatigue index in repeated 5-m shuttle run	12	85	9	85	5	12	86	8	80	12	No significant differences within- and between-groups
(Ouergui et al., 2020)^q^	fatigue index in repeated 5-m shuttle run	12	81	14	82	5	12	86	8	80	12	No significant differences within- and between-groups

^a^ : lower body training.

^b^ : upper-body training.

^c^ : Uchi-komi training.

^d^ : progressed HIIT.

^e^ : non-progressed HIIT.

^f^ : repeated sprint training.

^g^ ; 1:2 ratio.

^h^ ; 1:4 ratio.

^i^ : 1:8 ratio.

^j^ : technique specific.

^k^ ; 3:1 ratio.

^l^ : 2:1 ratio.

^m^ : kumi-kata.

^n^ : uchi-komi.

^o^ : running.

#: mean of the four bouts registered.

^p^ : repeated sprint training.

^q^ : repeated technique training.

In addition to power outputs, the studies on other outcomes, such as the SJFT index and KDI, showed mixed results. Some studies, like Franchini et al. (2016a) and Uchoa et al. (2020), found significant improvements post-HIIT, while others, such as Ouergui et al. (2022) and Ojeda-Aravena et al. (2021), observed no significant differences.

### 3.5 Meta-analysis

**FIGURE 2:**
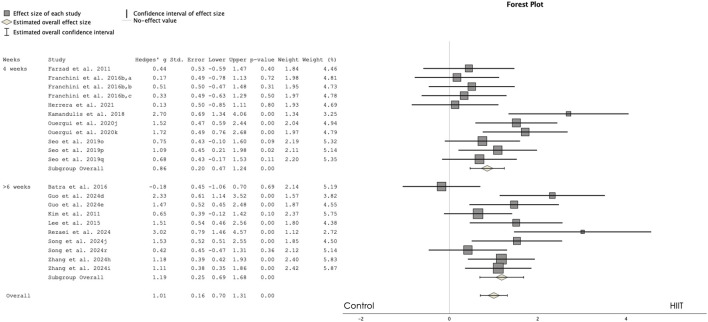
Forest plot showing the comparisons between high-intensity interval training (HIIT) and control groups in maximal oxygen uptake. a: lower body training; b: upper-body training; c: Uchi-komi training; d: progressed HIIT; e: non-progressed HIIT; j: repeated sprint training; o; 1:2 ratio; p; 1:4 ratio; q: 1:8 ratio; r: technique specific; s; 3:1 ratio j: repeated sprint training; k: repeated technique training.

Figure 2 presents a forest plot comparing the HIIT and control groups for VO2max. The results revealed a significant effect favoring HIIT over the control group, with a moderate magnitude of difference (ES = 1.007, 95% CI 0.701 to 1.312, p < 0.001, I^2^ = 54%). The analysis included 223 participants in the HIIT group and 222 participants in the control group, with the Egger test two-tailed result of 0.057.

Subgroup analysis comparing training programs of 4 weeks versus 6 weeks or more revealed that both were equally effective in enhancing aerobic performance compared to control groups. Specifically, studies with 4-week interventions showed significant moderate improvements (ES = 0.857, 95% CI 0.470 to 1.245, p < 0.001), while interventions of 6 weeks or more also promoted significant moderate improvements compared to control groups (ES = 1.185, 95% CI 0.690 to 1.680, p < 0.001).

**FIGURE 3:**
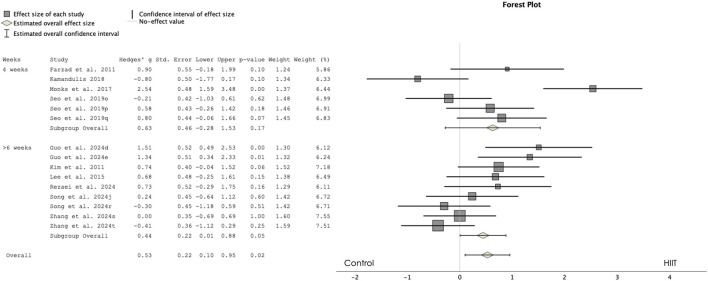
Forest plot showing the comparisons between high-intensity interval training (HIIT) and control groups in peak power output (PPO). d: progressed HIIT; e: non-progressed HIIT; j: repeated sprint training; o; 1:2 ratio; p; 1:4 ratio; q: 1:8 ratio; r: technique specific; s; 3:1 ratio t: 2:1 ratio.

Figure 3 presents a forest plot comparing the HIIT and control groups for PPO. The results revealed a significant effect favoring HIIT over the control group, with a small magnitude of difference (ES = 0.528, 95% CI 0.102 to 0.954, p = 0.015, I^2^ = 72%). The analysis included 170 participants in the HIIT group and 173 participants in the control group, with the Egger test two-tailed result of 0.46.

Subgroup analysis comparing training programs of 4 weeks versus 6 weeks or more revealed that only interventions of 6 weeks or more were significantly effective in improving PPO compared to control groups. Specifically, studies with 4-week interventions showed no significant differences from controls (ES = 0.629, 95% CI -0.277 to 1.535, p = 0.174), while interventions of 6 weeks or more resulted in significant small improvements compared to control groups (ES = 0.445, 95% CI 0.008 to 0.882, p = 0.046).

**FIGURE 4:**
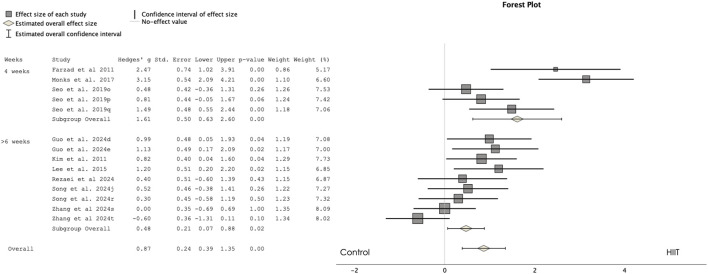
Forest plot showing the comparisons between high-intensity interval training (HIIT) and control groups in mean power output (MPO). d: progressed HIIT; e: non-progressed HIIT; j: repeated sprint training; o; 1:2 ratio; p; 1:4 ratio; q: 1:8 ratio; r: technique specific; s; 3:1 ratio t: 2:1 ratio; Supplementary Material 1. Search code in each database.

Figure 4 presents a forest plot comparing the HIIT and control groups for MPO. The results revealed a significant effect favoring HIIT over the control group, with a moderate magnitude of difference (ES = 0.871, 95% CI 0.392 to 1.350, p < 0.001, I^2^ = 75%). The analysis included 161 participants in the HIIT group and 174 participants in the control group, with the Egger test two-tailed result of 0.004.

Subgroup analysis comparing training programs of 4 weeks versus 6 weeks or more revealed that both were equally effective in enhancing MPO compared to control groups. Specifically, studies with 4-week interventions showed significant large improvements (ES = 1.615, 95% CI 0.628 to 2.602, p = 0.001), while interventions of 6 weeks or more also promoted significant small improvements compared to control groups (ES = 0.476, 95% CI 0.068 to 0.884, p = 0.022).

### 3.6 GRADE scale

Table 5 presents the certainty assessment for VO2max, PPO, and MPO outcomes using the GRADE framework. For all three outcomes, the evidence quality is rated as very low. This is mainly due to the high risk of bias in most of the included studies, as reflected by the downgrading of methodological quality. Moreover, significant inconsistency across studies (with I^2^ values of 54%, 71%, and 74%) further undermines the reliability of the results. The small sample sizes, each with fewer than 800 participants, contribute to the imprecision of the findings.

**TABLE 5:** GRADE analysis.

Outcomes (HIIT vs control)	Studies and PSS	Risk of bias in studies	Risk of publication bias	Inconsistency	Imprecision	Certainty of evidence
VO2max	13, n = 445	Downgrade by one level (good methodological quality)	No downgrade	Downgrade by one level (*I* ^2^ = 54%)	Downgrade by two levels: (i) <800 participants	⊕, Very low
Peak power output	10, n = 343	Downgrade by one level (good methodological quality)	No downgrade	Downgrade by one level (*I* ^2^ = 71%)	Downgrade by two level: (i) <800 participants	⊕, Very low
Mean power output	9, n = 335	Downgrade by one level (good methodological quality)	Downgrade by one level	Downgrade by one level (*I* ^2^ = 74%)	Downgrade by two levels: (i) <800 participants	⊕, Very low

i) *Risk of bias in studies*: downgraded by one level if good methodological quality in PEDro and two levels if poor; ii) *Indirectness*: considered low due to eligibility criteria; iii) *Risk of publication bias*: not assessed, as all comparison had <10 studies available; downgrade one level if Egger’s test <0.05; iv) *Inconsistency*: downgraded by one level when the impact of statistical heterogeneity (*I* ^2^) was moderate (>25%) and by two levels when high (>75%); v) *Imprecision*: downgraded by one level when <800 participants were available for a comparison or if there was no clear direction of the effects; accumulation of both resulted in downgrading by two levels.

GRADE: Grading of Recommendations Assessment, Development and Evaluation; HIIT: high-intensity interval training; PSS: pooled sample size.

## 4 Discussion

The results of this study suggest that HIIT provides significant performance benefits for athletes in Olympic combat sports. HIIT was found to improve aerobic capacity, peak power, and maximal power—key factors for success in combat settings. The findings indicate that HIIT enhances both endurance and anaerobic power, which are essential for the high-intensity, intermittent demands of combat sports. Overall, these results highlight that incorporating HIIT into training programs for combat athletes can lead to substantial improvements in these critical physical and physiological attributes, likely contributing to enhanced athletic performance.

The general trend across the reviewed studies indicates a positive response to HIIT interventions, with the majority showing improvements in VO2max from baseline to post-training measurements (Farzad et al., 2011; Batra and Zatoń, 2016; Ouergui et al., 2020, 2022; Herrera-Valenzuela et al., 2021; Guo and Mu, 2024). This observed increase aligns with the established benefits of HIIT for improving cardiorespiratory fitness in other sports (Engel et al., 2018). While the magnitude of VO2max enhancement varied across studies, likely due to methodological differences in HIIT protocols (training durations varied from 4 to 12 weeks), participant characteristics, and the specific combat sport engaged in, the overall tendency suggests that HIIT can effectively elicit favorable adaptations in this determinant physiological parameter. Additionally, the meta-analysis revealed a moderate effect size supporting the effectiveness of HIIT in comparison to control groups. Interestingly, similar effects were observed whether running or sport-specific techniques were used, as demonstrated in the study by Ouergui et al. (2020). These positive effects compared to control groups appear independent of training regimen, since one study showed that different work-to-rest ratios (1:2, 1:4, or 1:8) were all effective and significantly better than the control group (Seo et al., 2019).

Centrally, HIIT can drive increases in cardiac output through enhanced stroke volume and potentially heart rate modulation (Soeria Santoso and Boenyamin, 2019). Peripherally, HIIT promotes mitochondrial biogenesis and improves the efficiency of oxygen utilization within the muscle cells (Marques Neto et al., 2020). This enhanced mitochondrial function likely allows for greater rates of oxidative phosphorylation, contributing to a higher VO2max. Furthermore, HIIT may induce favorable changes in muscle fiber type distribution, shifting towards a greater proportion of oxidative fibers, which are more efficient at utilizing oxygen (Tan et al., 2018). Additionally, improvements in capillary density can further enhance oxygen delivery and removal of metabolic byproducts at the muscle level (Joyner and Casey, 2015).

Analysis of PPO data reveals a general trend towards improvement following HIIT interventions in combat sport athletes. The majority of studies exibited an increase in PPO from baseline to post-training. (Farzad et al., 2011; Kim et al., 2011; Monks et al., 2017; Kamandulis et al., 2018; Seo et al., 2019; Guo and Mu, 2024). While the meta-analysis confirmed this trend, the magnitude of the effect size was small. These findings suggest that HIIT can be an effective method for enhancing anaerobic power capabilities relevant to the demands of combat sports. These results were consistent regardless of HIIT periodization, as demonstrated by Guo and Mu (2024), who found that both progressive and non-progressive volume loading over the weeks led to improvements and significant differences compared to the control group. However, Seo et al. (2019) showed that PPO was only significantly greater than the control when using 1:4 and 1:8 work-to-rest ratios, while a 1:2 ratio was not as effective.

Improvements in the efficiency and capacity of the phosphagen system and glycolytic pathways can be a consequence of HIIT training, which are crucial for generating the ATP required for high-power activities (Abe et al., 2015). HIIT can also enhance the recruitment and synchronization of fast-twitch muscle fibers, which are responsible for generating maximal force and power (Vera-Ibañez et al., 2017). Furthermore, potential improvements in intramuscular buffering capacity may allow athletes to better tolerate the accumulation of metabolic byproducts (e.g., lactate) during high-intensity exercise, delaying fatigue and enabling greater power output (Forbes et al., 2008).

The delayed fatigue observed may also explain the improvements in MPO. HIIT’s effects on MPO mirrored the trend seen in PPO. The majority of studies reported significant improvements in MPO following HIIT interventions (Farzad et al., 2011; Monks et al., 2017; Kamandulis et al., 2018; Seo et al., 2019; Guo and Mu, 2024) with HIIT generally proving more effective than control or traditional combat training methods. This consistency across studies suggests that HIIT is a contributor to enhancing the capacity to sustain power output over time, a critical component of anaerobic performance in combat sports.

HIIT can improve lactate clearance, both through increased production of monocarboxylate transporters that shuttle lactate out of muscle cells and through enhanced activity of the lactate shuttle (Tamura et al., 2024). This improved clearance delays the accumulation of lactate and associated hydrogen ions (Bishop et al., 2008), which are key contributors to fatigue, allowing for the maintenance of higher power output for longer durations. Furthermore, while HIIT can also induce some mitochondrial adaptations, improving oxidative capacity (MacInnis and Gibala, 2017). While not the primary driver of MPO improvements, these adaptations play a supporting role, particularly in the recovery between high-intensity bouts and the ability to sustain repeated efforts.

### 4.1 Limitations

While this review suggests the potential benefits of HIIT for enhancing performance in Olympic combat sports, some limitations should be acknowledged. Variability in HIIT protocols across studies, including training duration, work-to-rest ratios, and specific exercise modalities, likely contributed to heterogeneity in aerobic and anaerobic outcomes, limiting definitive conclusions regarding optimal HIIT parameters. Moreover, most of studies showed a lack of blinding which may have influenced both participant performance and outcome assessment, potentially impacting the validity of reported effects. Beyond methodological concerns, this review highlights the need for further research to elucidate the physiological mechanisms by which HIIT exerts its effects on both aerobic and anaerobic performance in combat athletes. Future studies should incorporate measures of muscle fiber adaptations, metabolic responses, and neuromuscular changes to better understand how HIIT drives performance enhancements. Standardized HIIT protocols with blinding procedures are essential to strengthen the evidence base and minimize bias.

### 4.2 Practical implications

This review suggests that HIIT may improve aerobic capacity, peak and maximal power. These improvements are particularly relevant given the high-intensity, intermittent nature of combat sports. The consistent trend of increased VO2max, PPO and MPO across the reviewed studies, aligning with HIIT’s established benefits in other sports, highlights its effectiveness for enhancing cardiorespiratory fitness and anaerobic. While the magnitude of enhancement varied, likely due to methodological differences in study design, participant characteristics, and specific sport demands, the overall evidence suggests that HIIT elicits favorable adaptations in these measures. Similar performance gains were observed regardless of whether HIIT protocols utilized general exercises like running or sport-specific techniques, indicating flexibility in program design. This benefit appears independent of specific training regimen, as studies showed various work-to-rest ratios to be effective. Ultimately, the evidence strongly suggests that incorporating HIIT into training programs can lead to substantial improvements in the aerobic and anaerobic capacities necessary for enhanced athletic performance in Olympic combat sports.

## 5 Conclusion

The results of this review highlight the effectiveness of HIIT in improving key performance attributes for Olympic combat sport athletes. The findings consistently show significant enhancements in VO2max, PPO, and MPO, which are crucial for success in high-intensity, intermittent competition settings. While variations in training protocols influenced the magnitude of improvements, the overall trend supports HIIT as a beneficial training method. These performance gains were observed across different HIIT protocols, including both general and sport-specific exercises, exhibiting the versatility of HIIT in Olympic combat sports training. Given these findings, incorporating HIIT into training programs can be an effective strategy to enhance both endurance and anaerobic power, ultimately improving athletic performance.
