# Supplementary material for: Effects of high-intensity interval training on aerobic and anaerobic capacity in olympic combat sports: a systematic review and meta-analysis
Source: Front Physiol. 2025 May 9;16:1576676. doi: 10.3389/fphys.2025.1576676 (PMC12098572; doi:10.3389/fphys.2025.1576676)
Supplement: Supplementary file 1 [file Table1.docx]

**Supplementary material 1.** Search code in each database.

Pubmed (n=61)

(box*[Title/Abstract] OR wrestl*[Title/Abstract] OR judo[Title/Abstract] OR taekwondo[Title/Abstract] OR karate[Title/Abstract] OR fencing[Title/Abstract] OR fencer*[Title/Abstract]) AND ("high-intensity interval training"[Title/Abstract] OR "HIIT"[Title/Abstract] OR "intermittent exercise"[Title/Abstract] OR "sprint interval training"[Title/Abstract] OR "repeated sprint training"[Title/Abstract])

Scopus (n=156)

( TITLE-ABS-KEY ( box* OR wrestl* OR judo OR taekwondo OR karate OR fencing OR fencer* ) AND TITLE-ABS-KEY ( "high-intensity interval training" OR "HIIT" OR "intermittent exercise" OR "sprint interval training" OR "repeated sprint training" ) )

Web of Science (n=116)

box* OR wrestl* OR judo OR taekwondo OR karate OR fencing OR fencer* (Topic) and “high-intensity interval training” OR “HIIT” OR “intermittent exercise” OR “sprint interval training” OR “repeated sprint training” (Topic)
