# Supplementary material for: Effects of high-intensity interval training on aerobic and anaerobic capacity in olympic combat sports: a systematic review and meta-analysis
Source: Front Physiol. 2025 May 9;16:1576676. doi: 10.3389/fphys.2025.1576676 (PMC12098572; doi:10.3389/fphys.2025.1576676)
Supplement: Supplementary file 2 [file Table2.docx]

**Supplementary material 2.** Excluded studies in the full-text phase.

| Studies | Reason for exclusion |
| --- | --- |
| Haddad, M., Chaouachi, A., Wong del, P., Castagna, C., & Chamari, K. (2011). Heart rate responses and training load during nonspecific and specific aerobic training in adolescent taekwondo athletes. J Hum Kinet, 29, 59-66. doi:10.2478/v10078-011-0040-y | Analyzed only acute responses |
| Franchini, E., Panissa, V. L. G., & Julio, U. F. (2013). Physiological and performance responses to intermittent uchi-komi in judo. Journal of Strength and Conditioning Research, 27(4), 1147-1155. doi:10.1519/JSC.0b013e3182606d27 | Analyzed only acute responses |
| Bonato, M., Rampichini, S., Ferrara, M., Benedini, S., Sbriccoli, P., Merati, G., . . . La Torre, A. (2015). Aerobic training program for the enhancements of HR and VO2 off-kinetics in elite judo athletes. J Sports Med Phys Fitness, 55(11), 1277-1284. | No control group |
| Kazemi, A., Eslami, R., Ali, M. G., & Ghanbarzadeh, M. (2015). Effects of 6 weeks of low volume high intensity interval training on serum levels of leptin, glucose, and body fat in young wrestlers. Scientific Journal of Kurdistan University of Medical Sciences, 20(2), 70-77. | No aerobic or anaerobic outcomes |
| Branco, B. H. M., Lopes-Silva, J. P., Santos, J. F. D., Julio, U. F., Panissa, V. L. G., & Franchini, E. (2017). Monitoring training during four weeks of three different modes of high-intensity interval training in judo athletes. ARCHIVES OF BUDO, 13, 51-62. | No aerobic or anaerobic outcomes |
| Akhoundnia, K., Lamir, A. R., Khajeie, R., & Arazi, H. (2019). The effect of sport-specific High Intensity Interval Training on Ghrelin levels and body composition in youth wrestlers. Annals of Applied Sport Science, 7(1), 11-17. doi:10.29252/aassjournal.7.1.11 | No aerobic or anaerobic outcomes |
| Chacón Torrealba, T., Aranda Araya, J., Benoit, N., & Deldicque, L. (2020). Effects of High-Intensity Interval Training in Hypoxia on Taekwondo Performance. Int J Sports Physiol Perform, 15(8), 1125-1131. doi:10.1123/ijspp.2019-0668 | No comparator. Both groups were HIIT. |
| Ojeda-Aravena, A., Herrera-Valenzuela, T., Valdés-Badilla, P., Cancino-López, J., Zapata-Bastias, J., & García-García, J. M. (2021). Inter-Individual Variability of a High-Intensity Interval Training With Specific Techniques vs. Repeated Sprints Program in Sport-Related Fitness of Taekwondo Athletes. Front Physiol, 12, 766153. doi:10.3389/fphys.2021.766153 | No comparator. Both groups were HIIT. |
| Ojeda-Aravena, A., Herrera-Valenzuela, T., Valdés-Badilla, P., Martín, E. B., Cancino-López, J., Gallardo, J. A., . . . García-García, J. M. (2021). Effects of High-Intensity Interval Training With Specific Techniques on Jumping Ability and Change of Direction Speed in Karate Athletes: An Inter-individual Analysis. Front Physiol, 12, 769267. doi:10.3389/fphys.2021.769267 | No aerobic or anaerobic outcomes |
| Işık, B., Küççüktürk, S., Yüksel, M. F., Boyalı, E., Karaselek, M. A., & Erdağı, K. (2022). The effects of acute high intensity interval training on hematological parameters and neutrophils to lymphocytes ratio in elite taekwondo athletes according to gender. European Journal of Clinical and Experimental Medicine, 20(3), 306-315. doi:10.15584/ejcem.2022.3.8 | No control group |
| Kons, R. L., & Detanico, D. (2022). High-Intensity Interval Exercise Performance in Judo Athletes: Physiological, Perceptual, and Pacing Responses. Motor Control, 26(3), 353-361. doi:10.1123/mc.2022-0002 | Analyzed only acute responses |
| Yulfadinata, A., Setijono, H., Muhammad, H. N., Ayubi, N., & Kusnanik, N. W. (2022). High intensity interval training method potentially increases muscle strength in karate athletes. Journal of Physical Education and Sport, 22(12), 3051-3055. doi:10.7752/jpes.2022.12386 | No aerobic or anaerobic outcomes |
| Hagiwara, M., Yamagishi, T., Okamoto, S., Azuma, Y., & Yamashita, D. (2023). Short-term repeated sprint training in hypoxia improves explosive power production capacity and repeated sprint ability in Japanese international-level male fencers: A case study. Physiol Rep, 11(6), e15637. doi:10.14814/phy2.15637 | No control group |
| Kolimechkov, S., Makaveev, R., Zaykova, D., & Petrov, L. (2023). Tabata protocol-based high-intensity interval training in freestyle wrestlers. Pedagogy of Physical Culture and Sports, 27(6), 467-473. doi:10.15561/26649837.2023.0604 | No control group |
| Liu, H., & Li, Y. (2023). EFFECTS OF HIGH-INTENSITY INTERVAL TRAINING ON THE ANAEROBIC CAPACITY OF WRESTLERS. Revista Brasileira de Medicina do Esporte, 29. doi:10.1590/1517-8692202329012022_0279 | Compared athletes with non-athletes |
| Ouergui, I., Delleli, S., Messaoudi, H., Bridge, C. A., Chtourou, H., Franchini, E., & Ardigò, L. P. (2023). Repeated High-Intensity Technique Training and Repeated Sprint Training Elicit Similar Adjustment in Physiological Responses But Divergent Perceptual Responses and Combat-Related Performances in Adolescent Taekwondo Matches. Int J Sports Physiol Perform, 18(8), 825-832. doi:10.1123/ijspp.2022-0447 | No comparator. Both groups were HIIT. |
| Buzdagli, Y., Ozan, M., Baygutalp, N., Oget, F., Karayigit, R., Yuce, N., . . . Ucar, H. (2024). The effect of high-intensity intermittent and moderate-intensity continuous exercises on neurobiological markers and cognitive performance. BMC Sports Science, Medicine and Rehabilitation, 16(1). doi:10.1186/s13102-024-00831-7 | No aerobic or anaerobic outcomes |
| Kamandulis, S., Dudeniene, L., Snieckus, A., Kniubaite, A., Mickevicius, M., Lukonaitiene, I., . . . Stasiulis, A. (2024). Impact of Anaerobic Exercise Integrated into Regular Training on Experienced Judo Athletes: Running Vs. Repetitive Throws. Journal of Strength and Conditioning Research, 38(9), e489-e495. doi:10.1519/jsc.0000000000004829 | Counterbalanced |
| Usher, A., & Babraj, J. (2024). Impact of sprint interval training on post-fatigue mitochondrial rate in professional boxers. European Journal of Applied Physiology. doi:10.1007/s00421-024-05594-0 | No control group |
| Seo, M. W., Lee, J. M., Jung, H. C., Kim, J. Y., & Song, J. K. (2022). IDENTIFICATION OF THE OPTIMAL HIIT PROTOCOL FOR FATIGUE RESISTANCE IN ADOLESCENT ATHLETES: A RANDOMIZED CONTROLLED TRIAL. Kinesiology, 54(2), 256-267. doi:10.26582/k.54.2.3 | No aerobic or anaerobic outcomes |
| Hassan, A. K., Alibrahim, M. S., & Hammad, B. E. (2024). Influence of HIIT Training and Breathing Mask on Physiological, Biochemical Indicators, and Skill Performance in Taekwondo Players. International Journal of Human Movement and Sports Sciences, 12(5), 872-887. doi:10.13189/saj.2024.120513 | No randomized |
| Mañas-Paris, A., Muyor, J. M., & Oliva-Lozano, J. M. (2022). Using Inertial and Physiological Sensors to Investigate the Effects of a High-Intensity Interval Training and Plyometric Program on the Performance of Young Judokas. Sensors (Basel), 22(22). doi:10.3390/s22228759 | No randomized |
| Mischenko, N., Kolokoltsev, M., Gryaznykh, А., Vorozheikin, A., Romanova, E., & Suslina, I. (2021). Endurance development in Taekwondo according to the Tabata protocol. Journal of Physical Education and Sport, 21, 3162-3167. doi:10.7752/jpes.2021.s6421 | No randomized |
| Ravier G, Dugue B, Grappe F, et al. Impressive anaerobic adaptations in elite karate athletes due to few intensive intermittent sessions added to regular karate training. Scand J Med Sci Sports 19: 687–694, 2009. | No randomized |
